# Immune-mediated Colitis from Dual Checkpoint Inhibitors

**DOI:** 10.7759/cureus.6233

**Published:** 2019-11-26

**Authors:** Nishanth Thalambedu, Yasir Khan, Qian Zhang, Shristi Khanal, Ammar Ashfaq

**Affiliations:** 1 Internal Medicine, Abington Hospital - Jefferson Health, Abington, USA; 2 Internal Medicine, Abington Hospital, Jefferson Health, Abington, USA; 3 Internal Medicine, Abington Hospital-Jefferson Health, Abington, USA

**Keywords:** melanoma, immunotherapy, colitis, immune-related adverse events

## Abstract

Melanoma is a deadly disease with immunotherapy treatment options that emerged in the last few years and have changed the disease outcome. However, it is associated with immune-related toxic effects despite improving survival. We present the case of a 53-year-old woman who had two weeks of diarrhea after she was treated with dual immunotherapy agents for her advanced melanoma. The final workup revealed pancolitis, possibly due to immunotherapy adverse effects. Initial conservative treatment, unfortunately, did not lead to a clinical improvement until a steroid was introduced. We are reporting this case to alert our fellow physicians about the immune-mediated toxicities of the relatively new checkpoint inhibitors.

## Introduction

Immune checkpoint inhibitors emerged in the last decade, which has changed the disease outcomes of many types of cancers, with melanoma being one of them [1]. Anti-PD-1 antibody and anti-CTLA-4 antibody are currently approved as the first-line therapy for advanced melanoma. In spite of improving survival with these novel agents, they may sometimes have adverse gastrointestinal (GI) effects, ranging from mild diarrhea to severe colitis and even bowel perforation, which may lead to death. Our case presents an incident of severe worsening diarrhea and pancolitis while on dual immunotherapy despite being treated conservatively. Steroids were introduced due to the worsening of the condition after clinical improvement. The patient was discharged with a tapering dose of oral steroids. Consequently, the patient never received the same immunotherapy drugs and was switched to a different regimen.

## Case presentation

A 53-year-old female with a past medical history of advanced melanoma with metastasis to the brain and lungs presented to the hospital with a chief complaint of diarrhea that began two weeks ago. Diarrhea gradually progressed to the point where she was having 10-15 non-bloody bowel movements a day. She was getting treatment for melanoma with a combined nivolumab and ipilimumab immunotherapy. She finished her second cycle of therapy three weeks ago. She did endorse generalized abdominal pain. There were no fever, night sweats, or urinary complaints. On physical exam, she appeared dehydrated. The abdominal exam revealed generalized tenderness without guarding or rebound tenderness.

Laboratory evaluation included a comprehensive metabolic panel and complete blood count, which were normal except for a mild elevation of creatinine. Infectious workup, which included blood, urine, and stool cultures, were negative.

**Figure 1:**
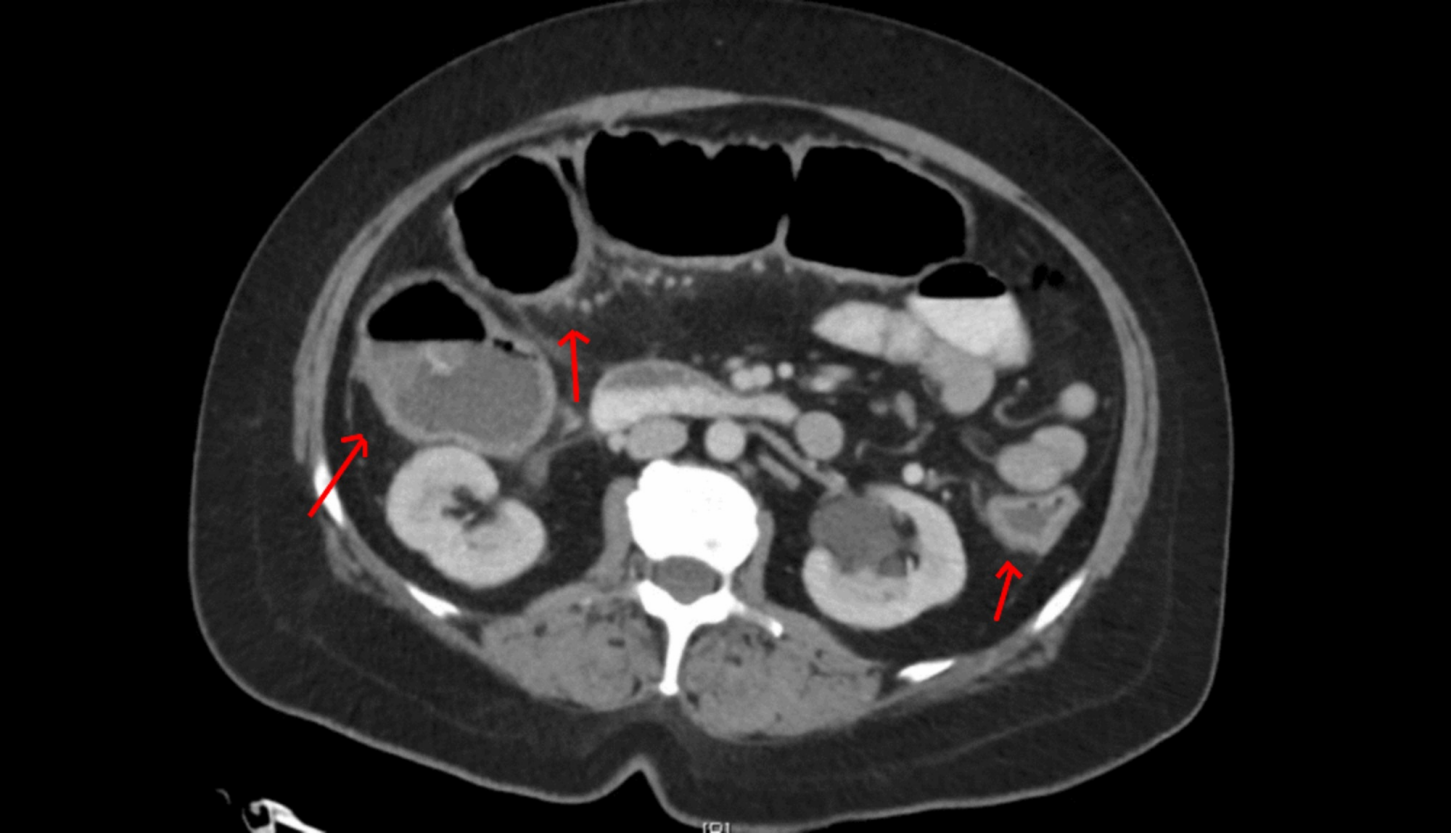
CT scan abdomen and pelvis Pancolitis

Radiological investigations included an abdominal X-ray, which did not reveal any obstruction, ileus, or free air. The patient also had a CT scan of the abdomen and pelvis with contrast that revealed pancolitis with no abscess (Figure 1).

The main differential diagnosis of her condition was between the infectious vs. inflammatory etiology of colitis. Her history and labs were more suggestive of a noninfectious etiology, likely due to immune=mediated toxicity due to the recent use of checkpoint inhibitors.

She was initially treated conservatively via intravenous fluids. No antibiotics were started. Her creatinine started to normalize with intravenous fluids. However, her symptoms failed to improve with conservative management, with a worsening of diarrhea. GI and surgery were consulted. The patient was started on intravenous dexamethasone 4 mg every six hours, which led to clinical improvement.

**Figure 2:**
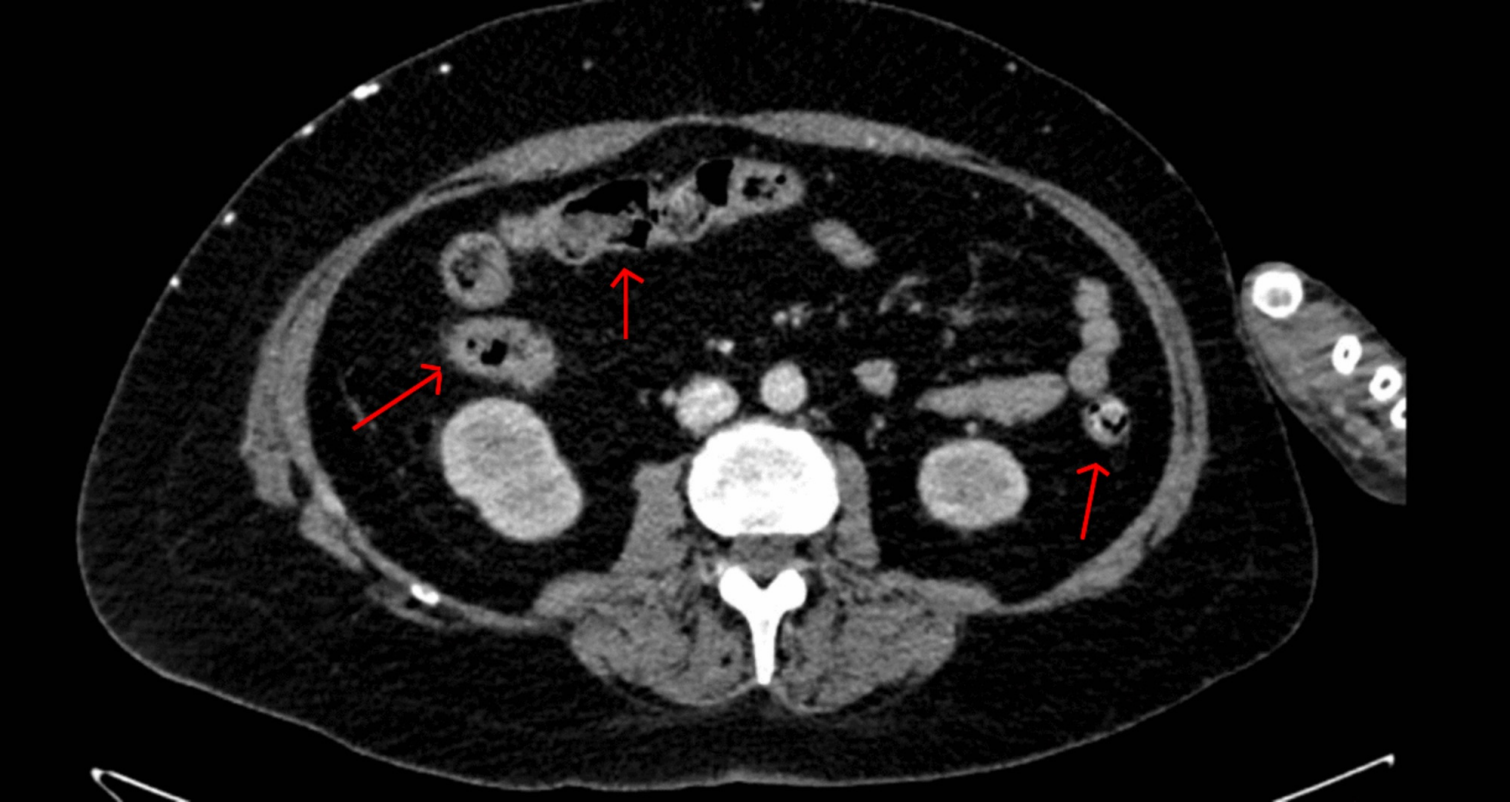
Repeat CT scan abdomen and pelvis Resolution of colitis

Her diarrhea started to improve. The diet was advanced and the patient was tolerating. Her dexamethasone was switched to oral prednisone 1 mg/kg. Ultimately, she was discharged on tapering doses of prednisone. Eventually, the patient was switched to Keytruda for her advanced melanoma that did not lead to any more adverse events. Her repeat CT scan showed resolution of colitis (Figure 2).

## Discussion

Melanoma is an aggressive malignancy arising from melanocytes [2]. It is a deadly disease that is reflected by an estimation of 96,000 new cases and around 8,000 deaths from metastatic melanoma in 2019 in the US [3].

The first-line treatment option with immunotherapy changed the fate of the disease by increasing progression-free survival (PFS) and overall survival (OS). The checkpoint inhibitors currently approved as the first line for metastatic melanoma are anti-CTLA-4 antibodies and anti-PD1 antibodies. They can be used as monotherapy or as combination therapy [4]. However, the use of these novel drugs are associated with immune-related toxicities. Gastrointestinal tract-related immune toxicities due to immunotherapy are also reported, with diarrhea and colitis being the most common [5-7].

The incidences of diarrhea and colitis were found to be 13.7% and 1.6%, respectively, with PD-1 inhibitors and 35.4% and 8.8% with CTLA-4 inhibitors. The mechanism of adverse events hypothesized is that the drugs may cause an alteration in normal cells and self-tolerance mechanisms, which results in T cell proliferation, leading to increased local cytokine release and symptoms manifestation [8].

The onset of adverse events is widely variable. It may occur between the first and tenth dose or any time frame up to 16 weeks within that of the last dose [9-11]. The relationship between the dose and adverse events has been studied with ipilimumab and showed increased adverse events with a higher dose [12]. Furthermore, the combination therapy of nivolumab and ipilimumab has shown to be associated with an increase in adverse effects than monotherapy alone [13]. In our case, the patient had dual immunotherapy with nivolumab and ipilimumab. These symptoms started after the second cycle of treatment.

The primary management of any immune-mediated toxicity involves discontinuing the culprit drug. Infectious causes of diarrhea must first be ruled out before considering treatment for immune-related adverse effects. Conservative management usually may resolve mild to moderate GI symptoms. Endoscopy with biopsy is usually recommended for symptoms that are severe or persistent. Other options include corticosteroids and anti-tumor necrosis factor (TNF) alpha agents [14-18]. Moreover, the use of prophylactic corticosteroids did not prove any reduction in the incidence of immune-related colitis and diarrhea and thus is not indicated [19]. Our patient had moderate to severe persistent symptoms that did not respond to conservative treatment. Clinical improvement was noticed after the initiation of steroid treatment. Anti-TNF alpha agents were not required.

## Conclusions

Immunotherapy has revolutionized cancer care for the majority of cancers, including melanoma. They are associated with immune-related adverse events with the majority of them being GI-related, with the possible symptoms of diarrhea and colitis. Anti-CTLA-4-based immunotherapies have more incidences of adverse events than anti-PD-1 based therapies. Dual immunotherapy is associated with more adverse events than monotherapy alone. The primary management involves discontinuing the drug and ruling out the infectious causes entity. Mild to moderate symptoms respond to conservative management. Severe and persistent symptoms require treatment with corticosteroids and possible biological agents. The prophylactic use of corticosteroids showed no benefit in reducing the adverse event rate. 
